# *Helicobacter pylori* Infection–Related Long Non-Coding RNA Signatures Predict the Prognostic Status for Gastric Cancer Patients

**DOI:** 10.3389/fonc.2021.709796

**Published:** 2021-07-27

**Authors:** Zhuoyuan Xin, Luping Zhang, Mingqing Liu, Yachen Wang, Yingli Zhang, Weidan Zhao, Yongxiao Sun, Lei Shi, Na Xu, Nan Zhang, Hong Xu

**Affiliations:** ^1^Department of Gastroenterology, The First Hospital of Jilin University, Changchun, China; ^2^The Key Laboratory of Zoonosis Research, Chinese Ministry of Education, College of Basic Medical Science, Jilin University, Changchun, China; ^3^Department of Gastrointestinal Colorectal and Anal Surgery, China-Japan Union Hospital of Jilin University, Changchun, China

**Keywords:** *Helicobacter pylori*, lncRNA, prognosis prediction, data mining, regulatory pattern

## Abstract

**Background:**

*Helicobacter pylori* (*H. pylori*) is a type I biological carcinogen, which may cause about 75% of the total incidence of gastric cancer worldwide. *H. pylori* infection can induce and activate the cancer-promoting signaling pathway and affect the occurrence and outcome of gastric cancer through controlling the regulatory functions of long non-coding RNAs (lncRNAs). However, we have no understanding of the prognostic worth of lncRNAs for gastric cancer patients infected with *H. pylori*.

**Method:**

We screened differentially expressed lncRNAs using DESeq2 method among TCGA database. And we built the *H. pylori* infection-related lncRNAs regulatory patterns. Then, we constructed *H. pylori* infection-based lncRNAs prognostic signatures for gastric cancer patients together with *H. pylori* infection, *via* uni-variable and multi-variable COX regression analyses. Based on receiver operator characteristic curve (ROC) analysis, we evaluated the prediction effectiveness for this model.

**Results:**

We identified 115 *H. pylori* infection–related genes were differentially expressed among *H. pylori*–infected gastric cancer tissues versus gastric cancer tissues. Functional enrichment analysis implies that *H. pylori* infection might interfere with the immune-related pathways among gastric cancer tissues. Then, we built *H. pylori* infection–related dys-regulated lncRNA regulatory networks. We also identified 13 differentially expressed lncRNAs were associated with prognosis for gastric cancer patients together with *H. pylori* infection. Kaplan-Meier analysis demonstrated that the lncRNA signatures were correlated with the poor prognosis. What is more, the AUC of the lncRNA signatures was 0.712. Also, this prognostic prediction model was superior to the traditional clinical characters.

**Conclusion:**

We successfully constructed a *H. pylori*–related lncRNA risk signature and nomogram associated with *H. pylori*–infected gastric cancer patients prognosis, and the signature and nomogram can predict the prognosis of these patients.

## Introduction

Gastric cancer (GC) is one of the most common cancers of gastrointestinal system. According to the world cancer report provided by World Health Organization (WHO), there are more than 1 million new cases of gastric cancer at 2018, together with 783,000 attributed deaths, which makes it the third leading cause of cancer-related mortality worldwide (1). *Helicobacter pylori* (*H. pylori*) is the type I biological carcinogens, which would cause nearly 75% of the total incidence of gastric cancer worldwide (2). Infection of *H. pylori* would induce or regulate the oncogenic metabolic pathways, and then affect the occurrences and outcomes of gastric cancer (3). So, *H. pylori* infection is considered to be one of the most serious risk factor and would affect the prognosis of gastric cancer patients. The identification of prognostic indicators complicated with the effects of *H. pylori* infection for GC patients is urgently needed.

Long noncoding RNAs (lncRNAs) are new kinds of linear functional RNAs, categorized by long sequence sizes (over 200 nucleotides), which are widely known to be involved in physiological and pathological regulations, such as cell cycle regulation, epigenetic regulation, as well as cell differentiations, and so on (4). Recently, lncRNAs are newly identified as gastric cancer regulators, which would control the activation or inhibition of cancer-related metabolic pathways caused by *H. pylori* infection (5). However, we have no understanding of the prognostic worth of lncRNAs for gastric cancer patients complicated with *H. pylori* infection.

Hence, in this study, we first collected *H. pylori* infection–related genes and identified the expression status, based on the RNA-Sequencing data provided by The Cancer Genome Atlas (TCGA) database. Then, we constructed the aberrant lncRNAs regulatory networks caused by *H. pylori* infection and extracted the epithelial cell signaling in *Helicobacter pylori* infection lncRNA regulatory pattern. And based on the lncRNAs profiles and clinical features, we built a multi-lncRNA prognostic signature for GC patients complicated with *H. pylori* infection that are correlated with poor prognosis. Also, this prognostic prediction model is superior to traditional clinical characters.

## Materials And Methods

### Data Collection

The RNA-sequencing data for 163 patients with *H. pylori* infection information were extracted from The Cancer Genome Atlas (TCGA) database, including lncRNA, miRNA, and mRNA profiles. We set up the gastric cancer tissues without *H. pylori* infection as a control group. Meanwhile, the clinical information of these patients was also obtained (**Table 1**). *H. pylori* infection–related genes were obtained from Gene Set Enrichment Analysis (https://www.gsea-msigdb.org/gsea/index.jsp) (**Figure S1**). Immunohistochemical images from the Human Protein Atlas (HPA) (https://www.proteinatlas.org) were used to identify the protein expression levels of five differentially expressed genes.

**Table 1:** Clinical Characters (N=163).

Characters	Counts
**Gender**	
Male	112
Female	51
***H. pylori* infection**	
Yes	18
No	145
**Age**	
≤65	95
>65	66
Not_Available	2
**T stage**	
T1	10
T2	36
T3	73
T4	44
**N stage**	
N0	38
N1	41
N2	40
N3	38
NX	5
Not_Available	1
**M stage**	
M0	146
M1	8
MX	9

### Construction of *H. pylori* Infection–Related lncRNAs Regulatory Patterns

Based on R version 4.0.3, “DESeq2” method was used to screen the differentially expressed lncRNAs, miRNAs, and mRNAs. Then, we used The Encyclopedia of RNA Interactomes (ENCORI) database (6) to construct *H. pylori* infection–related lncRNAs regulatory networks. We also used the STRING database to construct the PPI networks, and the networks were visualized by Cytoscape version 3.8.2.AB.

### Functional Enrichment Analysis

Based on R version 4.0.3, we used “clusterProfiler” package to perform gene ontology (GO) annotation and Kyoto Encyclopedia of Genes and Genomes (KEGG) enrichment analysis was also used. Then, we used “pheatmap”, “enrichplot”, “pathview”, and “ggplot2” packages to visualize the results.

### Screening *H. pylori* Infection–Related lncRNA Signatures

Lasso-penalized COX regression, univariate COX regression, and multivariate COX regression analyses were used to identify the *H. pylori* infection–related lncRNA signatures. Multivariate Cox regression analysis is based on the results of univariate regression analysis. The risk scores were calculated for each gastric cancer patients. And based on the risk scores, the patients were classified into high-risk (over median number) and low-risk (no more than median number) groups.

### The Nomogram

We used “rms” and “regplot” packages to construct the hybrid nomogram, based on R version 4.0.3. A nomogram was constructed integrating the prognostic signatures, for predicting 1-, 2-, and 3‐year OS of gastric cancer patients.

### Statistical Analysis

The significance of differences between comparative two groups was evaluated by Student’s *t*-test and the Wilcoxon test, respectively. Differences with P value less than 0.05 were considered significant. The data were analyzed using R version 4.0.3.

## Results

### Functional Analysis of *H. pylori* Infection–Related Differentially Expressed Genes in Gastric Cancer

**Figure 1:**
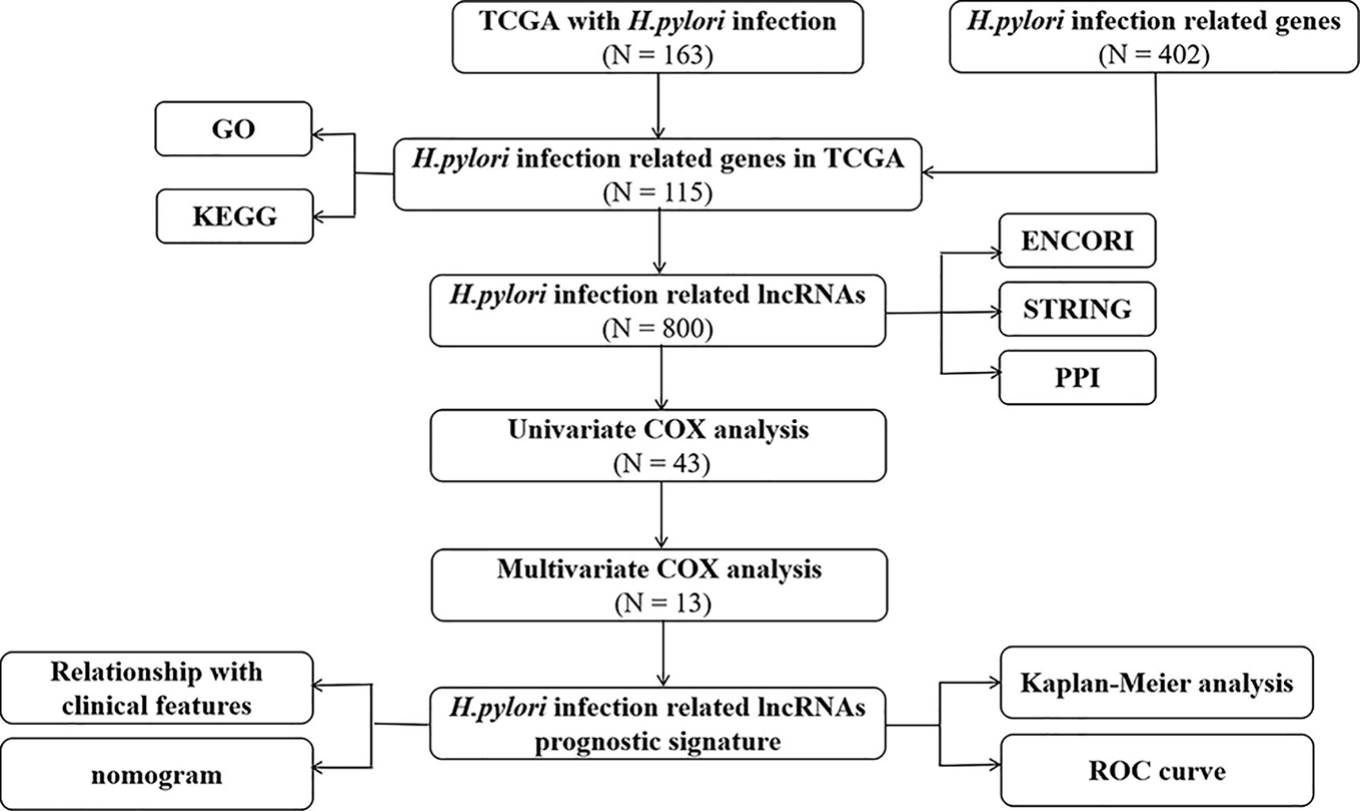
Flowchart of construction and validation of lncRNA signature and nomogram.

**Figure 2:**
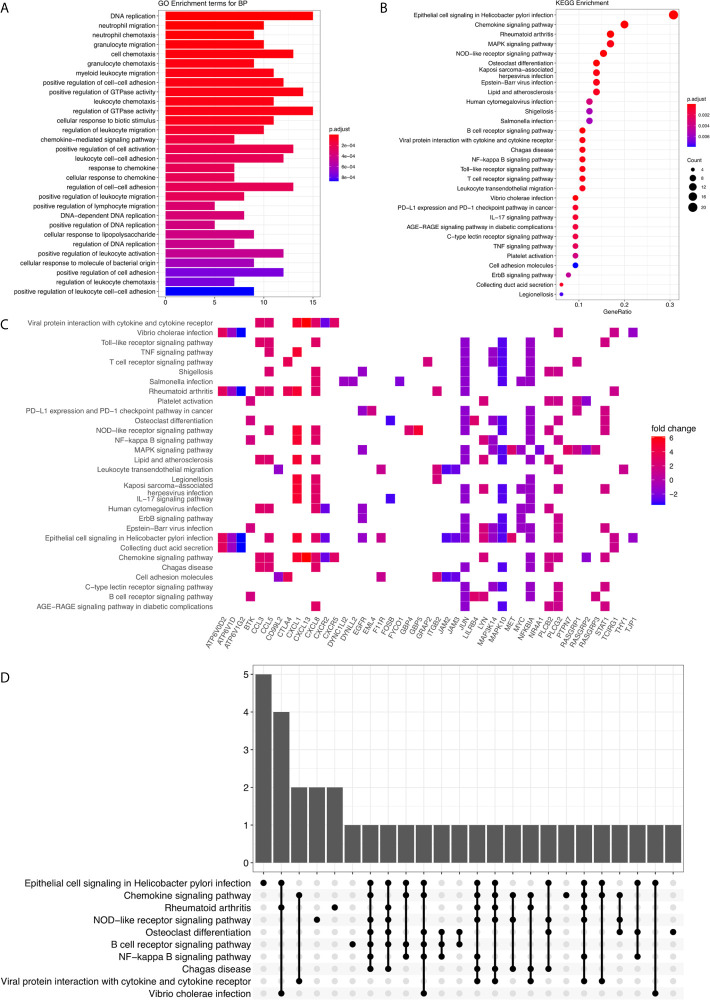
GO annotation and KEGG enrichment analysis. **(A)** The barplot for GO Biological Process terms. The x-axis represents the enriched gene counts. The bar colors represent the adjusted P-value. **(B)** The bubble diagram for KEGG terms. The x-axis represents the Gene Ratio. The dot colors represent the adjusted P-value. And the dot size represents the enriched gene counts. **(C)** The KEGG enrichment heatmap. **(D)** The KEGG enrichment upset plot.

The work flowchart for this study is shown in **Figure 1**. First, we have collected 402 *H. pylori* infection–related genes from Gene Set Enrichment Analysis (https://www.gsea-msigdb.org/gsea/index.jsp) (**Figure S1**) and identified 115 genes, which were differentially expressed among *H. pylori*–infected gastric cancer tissues (among which 77 were up-regulated and 38 were down-regulated; **Figure S2**). We selected five genes that are significantly differentially expressed and searched their protein expression levels from the Human Protein Atlas (HPA). Immunohistochemical images from HPA indicated high levels of GBP5, ATP6V1G2 protein in gastric cancer tissues and GBP5, ATP6V1G2 protein were not detected in normal stomach tissues (**Figures S2A** **–D**). However, the expression levels of CXCL8 are opposite (**Figures S2E, F**). In addition, HPA indicated low levels of DEPDC1 protein in normal stomach tissues and moderate expression level in gastric cancer tissues (**Figures S2G, H**); however, the expression levels of MET are the opposite (**Figures S2I, J**). GO Biological Process annotation identified that these genes participated in “GO:0071219 Cellular response to molecule of bacterial origin,” “GO:0002685 Regulation of leukocyte migration,” as well as “GO:0007159 Leukocyte cell-cell adhesion” (**Figure 2A** and **Table S3**). Following which, KEGG enrichment analysis has shown that these aberrantly expressed genes were significantly involved in “hsa05120: Epithelial cell signaling in *H. pylori* infection” and “hsa04061: Viral protein interaction with cytokine and cytokine receptor” (**Figure 2B** and **Table S4**). Furthermore, *H. pylori* infection–related oncogenic signaling pathways, including “hsa04064: NF-kappa B signaling pathway” and “hsa04010: MAPK signaling pathway,” were also significantly enriched (**Figures 2C, D** and **S1**). So, these items imply that the *H. pylori* infection can induce and activate the cancer-promoting signaling pathway and affect the occurrence and outcome of gastric cancer.

### The *H. pylori* Infection–Related lncRNAs Regulatory Patterns in Gastric Cancer

**Figure 3:**
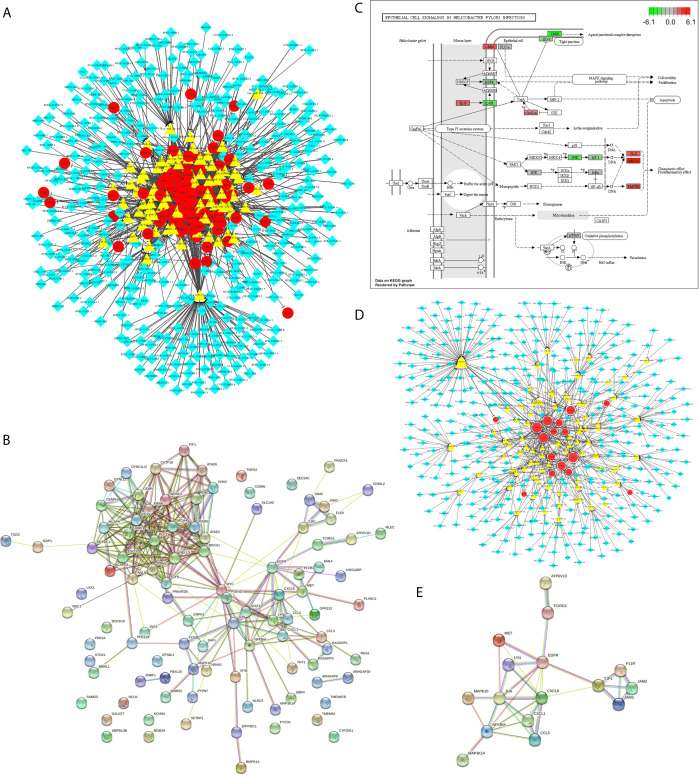
The *H. pylori* infection–related lncRNAs regulatory patterns. **(A)** The aberrant lncRNA regulatory networks among *H. pylori*–infected gastric cancer tissues. The red circles represent the differentially expressed *H. pylori* infection–related genes. The yellow triangles represent the miRNAs. And the blue diamonds represent the lncRNAs. **(B)** PPI network for *H. pylori* infection–related genes involved in A. **(C)** KEGG graph for “hsa05120: Epithelial cell signaling in Helicobacter pylori infection”. The colored molecules were enriched differentially expressed *H. pylori* infection–related genes. The color represents the log2 (Fold Change) values for each molecule. Red represents the up regulation. Green represents the down regulation. **(D)** The Epithelial cell signaling in *H. pylori* infection lncRNA regulatory pattern. The red circles represent the differentially expressed *H. pylori* infection–related genes. The yellow triangles represent the miRNAs. And the blue diamonds represent the lncRNAs. **(E)** PPI network for target genes involved in Epithelial cell signaling in *H. pylori* infection lncRNA regulatory pattern.

Then, we have identified 800 differentially expressed lncRNA among *H. pylori*–infected gastric cancer tissues (among which 443 were up-regulated and 357 were down-regulated, **Table S5**). Using The Encyclopedia of RNA Interactomes (ENCORI) database (6), we constructed the *H. pylori* infection–related lncRNAs regulatory network (**Figure 3A** and **Table S6**), based on the 115 aberrant expressed *H. pylori* infection–related genes we just identified. This network contains 370 lncRNAs, 92 miRNAs, and 100 genes. Using STRING database version 11.0, the protein-protein interaction (PPI) relationships for the 100 target genes were constructed (**Figure 3B** and **Table S7**). Next, based on the KEGG enrichment items, we mapped the genes on hsa04064 KEGG graph (**Figure 3C**) and constructed the “Epithelial cell signaling in *H. pylori* infection lncRNA regulatory pattern” (**Figure 3D** and **Table S8**). Furthermore, we also identified the PPI relationships for the genes involved in this lncRNA regulatory pattern (**Figure 3E** and **Table S9**).

### The *H. pylori* Infection–Based lncRNAs Prognostic Signature

To analyze the correlation between *H. pylori* infection–related lncRNAs and prognosis, we used univariate COX analysis and identified 43 significant *H. pylori* infection (**Table 2**), which would be used in multivariate COX analysis (**Table 3**). In summary, we found that 13 aberrantly expressed lncRNAs may be independent prognosis prediction factors for *H. pylori*–infected gastric cancer (including AC007319.1, AP000593.7, LINC00304, LINC01526, RP11-197P3.5, RP11-284F21.7, RP11-319E16.2, RP11-334C17.5, RP11-617F23.2, RP11-649G15.2, RP11-706O15.3, RP11-867G23.12, as well as RP4-791M13.3, **Table 3**). Also, the risk scores were also calculated to construct a prognostic signature for *H. pylori*–infected gastric cancer (**Table S10**).

**Table 2:** Univariate COX regression analysis with the Lasso method for the *H. pylori* infection–related lncRNA prognostic signature.

lncRNAs	HR	HR.95L	HR.95H	P Value
**AC003090.1**	1.00232658	1.00060336	1.00405278	0.0081206
**AC006273.4**	1.01108259	1.00187346	1.02037637	0.01823098
**AC007319.1**	1.00994767	1.00290495	1.01703985	0.00556424
**AP000593.7**	1.01204229	1.00325146	1.02091015	0.00716133
**BOLA3-AS1**	1.00157614	1.0000171	1.0031376	0.04753716
**CPEB2-AS1**	1.04596159	1.00126671	1.09265159	0.04371864
**CTB-102L5.7**	1.00473681	1.00074407	1.00874548	0.0200144
**DPH6-AS1**	1.00923141	1.00021005	1.01833415	0.04487659
**ERVMER61-1**	1.00342091	1.0008008	1.00604789	0.01046663
**FLG-AS1**	1.00668347	1.00222755	1.0111592	0.00325009
**HOTTIP**	0.99948013	0.99901721	0.99994326	0.02780426
**KCNMA1-AS1**	1.01293373	1.00110354	1.02490373	0.03203517
**LINC00304**	1.01785079	1.00689614	1.02892463	0.00135174
**LINC00461**	1.01443178	1.00194427	1.02707493	0.02337065
**LINC00494**	1.00098569	1.00013723	1.00183488	0.02277914
**LINC01356**	0.99365549	0.98789476	0.99944982	0.03191488
**LINC01526**	1.17352838	1.07697987	1.27873223	0.00025922
**RNF144A-AS1**	1.0028236	1.00001509	1.00563999	0.04877969
**RP11-103B5.4**	1.00069073	1.00008856	1.00129326	0.0245556
**RP11-1103G16.1**	1.00450826	1.00064783	1.00838359	0.0220439
**RP11-1134I14.8**	1.00740109	1.00162968	1.01320576	0.01188814
**RP11-119F7.5**	1.00284554	1.00014366	1.00555472	0.03898734
**RP11-169F17.1**	1.00028572	1.00001013	1.0005614	0.04215272
**RP11-197P3.5**	0.99067852	0.98201334	0.99942017	0.03667523
**RP11-284F21.10**	0.99975668	0.99954068	0.99997273	0.0272903
**RP11-284F21.7**	0.99906916	0.99835054	0.99978829	0.01118991
**RP11-314A20.2**	1.00734424	1.00045068	1.0142853	0.03674647
**RP11-319E16.2**	1.00632252	1.00236789	1.01029276	0.00170558
**RP11-334C17.5**	1.00100398	1.00018373	1.00182491	0.01643073
**RP11-346D19.1**	1.01072463	1.00280024	1.01871163	0.0079012
**RP11-378J18.8**	1.00274126	1.00007305	1.00541659	0.04404136
**RP11-395G23.3**	1.00617622	1.0002114	1.01217661	0.04239286
**RP11-43F13.3**	1.00267629	1.00044035	1.00491723	0.01895186
**RP11-617F23.2**	1.00438034	1.00069084	1.00808344	0.01992498
**RP11-649G15.2**	0.97526816	0.95327835	0.99776522	0.03137831
**RP11-706O15.3**	0.99764769	0.99591353	0.99938487	0.00797425
**RP11-736K20.5**	1.00559473	1.00006226	1.0111578	0.04746999
**RP11-826N14.2**	1.07617911	1.01767605	1.13804532	0.01004258
**RP11-867G23.12**	0.95950777	0.93068014	0.98922833	0.00791182
**RP3-522D1.1**	0.99286591	0.98663244	0.99913876	0.02587385
**RP4-737E23.2**	1.01471489	1.00267716	1.02689715	0.01643757
**RP4-791M13.3**	1.18477054	1.02826137	1.36510159	0.01900145
**RP5-1085F17.3**	1.00055999	1.00009364	1.00102655	0.01859089

Cl, confidence interval; HR, hazard ratio.

**Table 3:** Multivariate COX regression analysis with the Lasso method for the 13 *H. pylori* infection–related lncRNA prognostic signature.

lncRNAs	coef	HR	HR.95L	HR.95H	P Value
**AC007319.1**	0.00719639	1.00722234	0.99895525	1.01555785	0.08701075
**AP000593.7**	0.01068028	1.01073752	1.00000475	1.02158548	0.04989821
**LINC00304**	0.01429421	1.01439686	1.00047933	1.028508	0.04256517
**LINC01526**	0.08924022	1.09334326	0.9995469	1.19594137	0.05116888
**RP11-197P3.5**	−0.0131777	0.98690874	0.97640213	0.9975284	0.01581625
**RP11-284F21.7**	−0.0009948	0.99900574	0.9981591	0.9998531	0.0214726
**RP11-319E16.2**	0.0066417	1.0066638	1.00237916	1.01096676	0.00227393
**RP11-334C17.5**	0.00135657	1.00135749	1.00038945	1.00232647	0.00597758
**RP11-617F23.2**	0.0032673	1.00327264	0.99926059	1.00730081	0.11000806
**RP11-649G15.2**	−0.0411068	0.95972667	0.93236029	0.98789631	0.00535283
**RP11-706O15.3**	−0.0017858	0.99821577	0.99660249	0.99983167	0.03046813
**RP11-867G23.12**	−0.049286	0.95190881	0.91804723	0.98701935	0.00765377
**RP4-791M13.3**	0.18462453	1.20276675	1.0043068	1.44044415	0.04478441

Cl, confidence interval; HR, hazard ratio.

### Survival Analysis Based on High-Risk lncRNA Signatures

**Figure 4:**
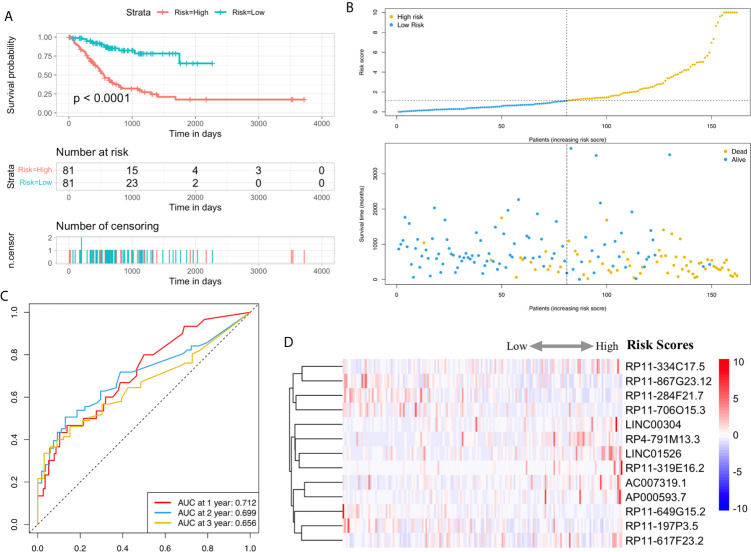
H. pylori infection–related high-risk lncRNA signature. **(A)** The Kaplan-Meier curves. **(B)** The risk survival status plot. **(C)** The ROC curves for the prediction of 1-, 2-, and 3-year survival rate of gastric cancer. **(D)** Heatmap for the risk scores.

Utilizing Kaplan-Meier analysis, we identified that the high-risk lncRNA signatures were associated with poor survival condition (*P* value < 0.0001, **Figure 4A**). And the patient risk survival status plot demonstrated that the risk-scores were proportional to the survival of patients inversely (**Figure 4B**). Using ROC curve plotting, it was present that the AUC values for the high-risk lncRNA signatures at 1-, 2-, and 3-year survival rates were 0.712, 0.699, and 0.656, respectively (**Figure 4C**). It was a heatmap for the risk scores (**Figure 4D**).

### COX Analysis for the Expression of *H. pylori* Infection–Related lncRNA Signatures

**Figure 5:**
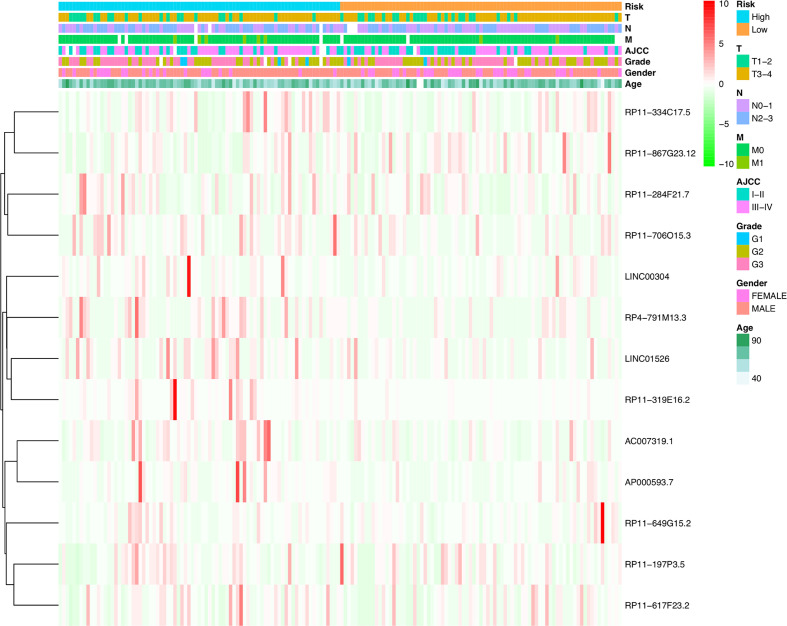
Heatmap for *H. pylori* infection–related lncRNAs prognostic signature and clinical features.

**Figure 6:**
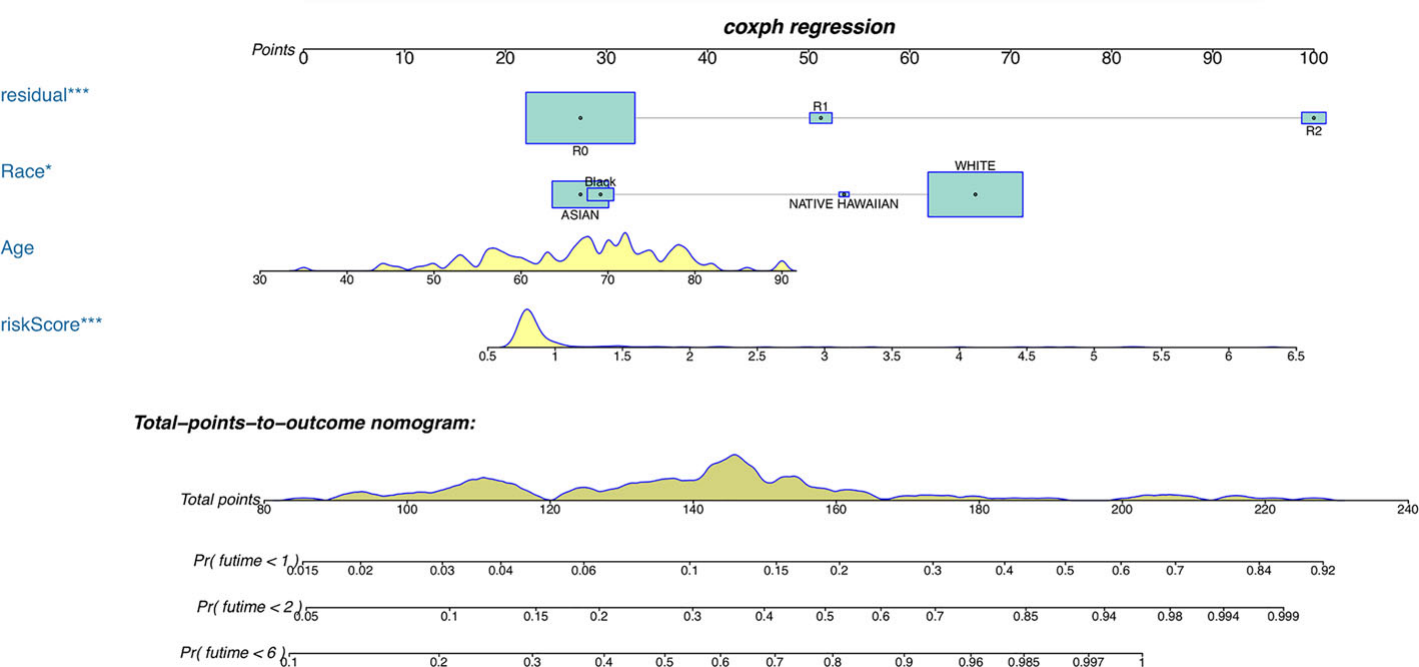
A nomogram for both clinical features and *H. pylori* infection–related lncRNAs prognostic signatures. *p < 0.05, ***p < 0.001.

Furthermore, COX analysis results have shown that the *H. pylori* infection–related lncRNA signatures (HR, 1.401; 95% CI, 1.243–1.579), AJCC stages (HR, 2.311; 95% CI, 1.293–4.129) as well as TNM stages were independent prognosis factors of OS of *H. pylori*–infected gastric cancer patients (**Table 4**). Also, the correlations among *H. pylori* infection–related lncRNA prognostic signatures and clinical factors were also exhibited using heatmap (**Figure 5**). Finally, using hybrid nomogram, which merged the clinical characteristics, we verified that the *H. pylori* infection–related lncRNA prognostic signatures were accurate and relatively stable (**Figure 6**). Hence, these lncRNA signatures may be used in the prognosis evaluation for *H. pylori*–infected gastric cancer patients.

**Table 4:** COX analysis for clinical factors.

	HR	HR.95L	HR.95H	P Value
**Risk score**	1.40074826	1.24281815	1.57874722	3.36E-08
**Age**	1.02930064	1.00325806	1.05601924	0.02719286
**Gender**	1.59499434	0.91761675	2.77240684	0.09789177
**Grade**	2.09599018	0.28389267	15.4747736	0.46814054
**Race**	33.8853803	3.38021215	339.688442	0.00273934
**AJCC**	2.31094767	1.29337404	4.12910647	0.00467395
**M**	1.71967673	0.68721196	4.30331284	0.246689
**N**	1.88998085	1.14261245	3.12619352	0.01316835
**T**	1.99362375	1.04165621	3.81559254	0.03723408

## Discussion

The major risk factor associated with gastric adenocarcinoma is infection by *H. pylori*, and the attributable proportion of *H. pylori*–infected gastric cancer patients has been estimated to be about 70% (7). Thus, it is particularly critical to explore accurate biomarkers for predicting prognosis of *H. pylori*–infected gastric cancer patients. In this study, we first identified a novel 13-lncRNA risk signature that was highly associated with the OS of *H. pylori*–infected gastric cancer patients based on the TCGA.

Overall, we identified 115 *H. pylori*–related differentially expressed genes in GC patients. KEGG analyses further revealed that the differentially expressed genes mainly participated in GO and identified that these genes participated in the cellular response to molecule of bacterial origin, regulation of leukocyte migration, and leukocyte cell-cell adhesion. KEGG analyses revealed that the genes mainly participated in epithelial cell signaling in *H. pylori* infection, viral protein interaction with cytokine and cytokine receptor, NF-kappa B signaling, and MAPK signaling pathway. So, these items imply that the *H. pylori* infection can induce and activate the cancer-promoting signaling pathway and affect the occurrence and outcome of gastric cancer. In addition, we also identified 13 differentially expressed lncRNAs associated with prognosis for gastric cancer patients together with *H. pylori* infection. Among them, Zhang et al. indicated that LINC00304 plays a tumor-promotive functional role and promotes PCa cell proliferation and cell cycle by upregulating CCNA1 expression (8). Cheng et al. showed that LINC01526 was among the noninvasive prognostic signatures, which was a preoperative prediction of disease-free survival in GC patients (9). However, the molecular mechanism and effect of other lncRNAs in our 13-lncRNA signature have not been explored and are still uncertain. Increasing studies showed that lncRNAs play important roles in tumorigenesis and metastasis by regulating the expression of protein-coding genes through transcriptional, posttranscriptional, and posttranslational regulation (10–13). Also, several reports indicated that LncRNAs may show oncogenic and tumor-suppressive activities in the initiation and progression of GC (14). Yang et al. reported that LINC00152 and H19 were significantly increased in the serum and cancer tissues of patients with GC and may be possible biomarkers for diagnosis and prognosis of GC (15). Another study showed that non-overlapping signatures of a few lncRNAs were abnormally expressed in gastric epithelial cells infected by *H. pylori*, and these lncRNAs may have a function in the immune system response against *H. pylori*, which in turn may lead to the occurrence of gastric cancer with *H. pylori* (16). Several studies indicated the influence of *H. pylori* infection on the dysregulation of lncRNA expression profiles, including Lnc-SGK1, THAP9-AS1, NR-026827, and AF147447 (17–20).

Here, the differentially expressed *H. pylori*–associated lncRNAs in our study were stratified into two groups of high- and low-risk scores to explore their potential roles in *H. pylori*–infected gastric cancer patients. Our study indicated that patients with H. pylori infection in the high-risk group had a shorter survival than those in the low-risk group, and the signature also displayed a high prediction sensitivity and specificity. In this study, we constructed a nomogram by combining the 13-lncRNA signature and clinicopathological factors in 162 patients from the TCGA, and the nomogram is an effective predictive tool for patients with *H. pylori* infection.

A large number of studies have explored the prognostic factors of gastric cancer from the level of genes and proteins. The expression of HOXC9 mRNA and HOXC9 protein in gastric cancer tissue was significalntly higher than that in non-gastric cancer tissue (21). CD40, CAP2 proteins, and mRNAs expressions are closely related to prognosis, distant metastasis, and stage of patients with gastric cancer (22, 23). The overall survival rate of patients with up-regulated SLC22A16 expression was worse than that of patients without up-regulated SLC22A16 expression (24). CXCR7 may be a prognostic marker for gastric cancer with peritoneal metastasis; however, the number of cases in this study were small, and the prognostic significance of CXCR7 was not confirmed (25). Wang et al. indicated that the high expression of SCD1 might predict poor prognosis in gastric cancer patients, but the AUC value at 1-, 3-, and 5-year survival rates were 0.557, 0.569, and 0.595, respectively (26), were significantly lower than the results of our study. In addition, the AUC’s value of another predictive biomaker (linc-ROR) was 0.6495 (27), which was also lower than ours. So, we proposed that our findings improved the prediction accuracy for the prognosis of gastric cancer patients.

*H. pylori* is considered as a precancerous lesion of gastric cancer and is closely related to the occurrence and development of gastric cancer (28). The eradication of *H. pylori* can reduce the incidence of gastric cancer (29, 30), but the prevalence of *H. pylori* is high, especially in economically underdeveloped areas (31). It has been reported that the prognostic factors of patients with gastric cancer infected with *H. pylori*, for example, miR-490-3p is associated with poor clinical prognosis. Low expression of miR-490-3p is associated with poor prognosis in *H. pylori*–infected gastric cancer patients. However, the molecular functions of miR-490-3p are very diverse, and miR-490-3p can also promote the development of some cancers. The function of miR-490-3p is closely related to the tumor microenvironment (32). The expression levels of anti-*H. pylori* antibody, CA724, CA19-9, and CEA in *H. pylori*–infected gastric cancer patients were positively correlated with tumor stage, tumor size, and lymph node (33).However, Tas and Chae pointed out that the levels of CA724, CA19-9, CEA, and other markers had no significance in evaluating the prognosis of gastric cancer patients (34, 35). In addition, this study was a retrospective study with a small sample size and the same hospital. Therefore, the accuracy of anti-*H. pylori* antibody, CA724, CA19-9, and CEA in predicting *H. pylori*–infected gastric cancer patients is still to be discussed. DNA methylation can induce the occurrence of gastric cancer in *H. pylori*–infected gastric cancer patients (36). However, this study did not establish a prediction model and could not accurately predict the patients.

Our research has some obvious strengths. First, the effect of lncRNA on the prognosis of *H. pylori*–infected gastric cancer patients has never been reported, and our research is the first to present this. Second, in this study, a model affecting the prognosis of *H. pylori*–infected gastric cancer patients was established based on the level of lncRNA, and a nomogram was constructed, which is conducive to more accurate prediction of the prognosis of *H. pylori*–infected gastric cancer patients and improvement of the survival rate of patients. Also, the signature and nomogram is convenient to apply in clinical practice for doctors. In addition, there are several limitations. First, the number of patients included in the study is relatively insufficient. Second, the 13-lncRNA signature and predicted nomogram were not validated in both internal and external cohorts and not compared with other models. Third, there is still a lack of experimental researches about the molecular function of these lncRNAs, and we will collect specimens from *H. pylori* (+) and *H. pylori* (−) GC patients and carry out experimental research.

In conclusion, we successfully constructed an *H. pylori*–related lncRNA risk signature and nomogram associated with *H. pylori*–infected gastric cancer patients prognosis in TCGA. The results indicated that the signature and nomogram can predict the prognosis of these patients. Furthermore, we should carry out related molecular research to clarify the mechanism of related lncRNA in the future.

## Data Availability Statement

The original contributions presented in the study are included in the article/**Supplementary Material**. Further inquiries can be directed to the corresponding authors.

## Author Contributions

ZX, NZ, and HX came up with the design and critical revision of the manuscript. The data analysis were conducted by YW, YZ, YS, and WZ. The original writing of the draft and its editing were conducted by LZ, ML, LS, and NX. All authors contributed to the article and approved the submitted version.

## Funding

This work was supported by the Department of Finance of Jilin Province (JLSWSRCZX2020-083), Department of Science and technology of Jilin Province (3D517DF53428, 2020Q051), and the Bethune Project of Jilin University (2020B09).

## Conflict of Interest

The authors declare that the research was conducted in the absence of any commercial or financial relationships that could be construed as a potential conflict of interest.

## Publisher’s Note

All claims expressed in this article are solely those of the authors and do not necessarily represent those of their affiliated organizations, or those of the publisher, the editors and the reviewers. Any product that may be evaluated in this article, or claim that may be made by its manufacturer, is not guaranteed or endorsed by the publisher.

## Supplementary Material

Supplementary Figure 1 KEGG graph. **(A)** KEGG graph for “hsa04064: NF-kappa B signaling pathway” **(B)** KEGG graph for “hsa04010: MAPK signaling pathway. The colored molecules were enriched differentially expressed *H. pylori* infection–related genes. The color represents the log2 (Fold Change) values for each molecule. Red represents the up regulation. Green represents the down regulation.

Supplementary Figure 2 Immunohistochemical images from HPA. **(A, B)** The immunohistochemical images of GBP5 in normal stomach tissues and gastric cancer tissues. **(C, D)** The immunohistochemical images of ATP6V1G2 in normal stomach tissues and gastric cancer tissues. **(E, F)** The immunohistochemical images of CXCL8 in normal stomach tissues and gastric cancer tissues. **(G, H)** The immunohistochemical images of DEPDC1 in normal stomach tissues and gastric cancer tissues. **(I, J)** The immunohistochemical images of MET in normal stomach tissues and gastric cancer tissues.
