# Supplementary figures and images for: Helicobacter pylori Infection–Related Long Non-Coding RNA Signatures Predict the Prognostic Status for Gastric Cancer Patients
Source: Front Oncol. 2021 Jul 27;11:709796. doi: 10.3389/fonc.2021.709796 (PMC8353258; doi:10.3389/fonc.2021.709796)

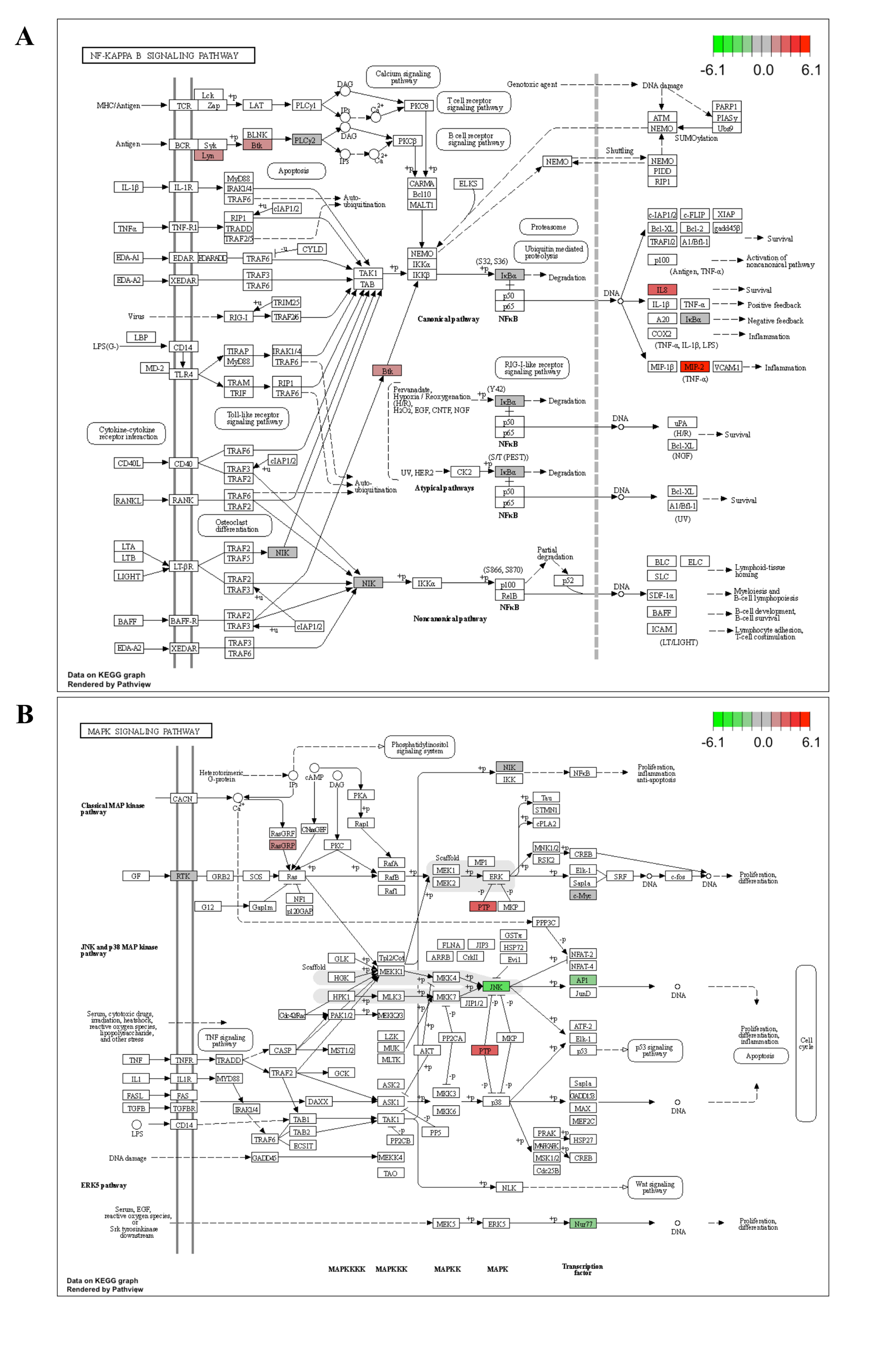

Supplement: Supplementary Figure 1 — KEGG graph. (A) KEGG graph for “hsa04064: NF-kappa B signaling pathway” (B) KEGG graph for “hsa04010: MAPK signaling pathway. The colored molecules were enriched differentially expressed H. pylori infection–related genes. The color represents the log2 (Fold Change) values for each molecule. Red represents the up regulation. Green represents the down regulation. [file Image_1.tif]

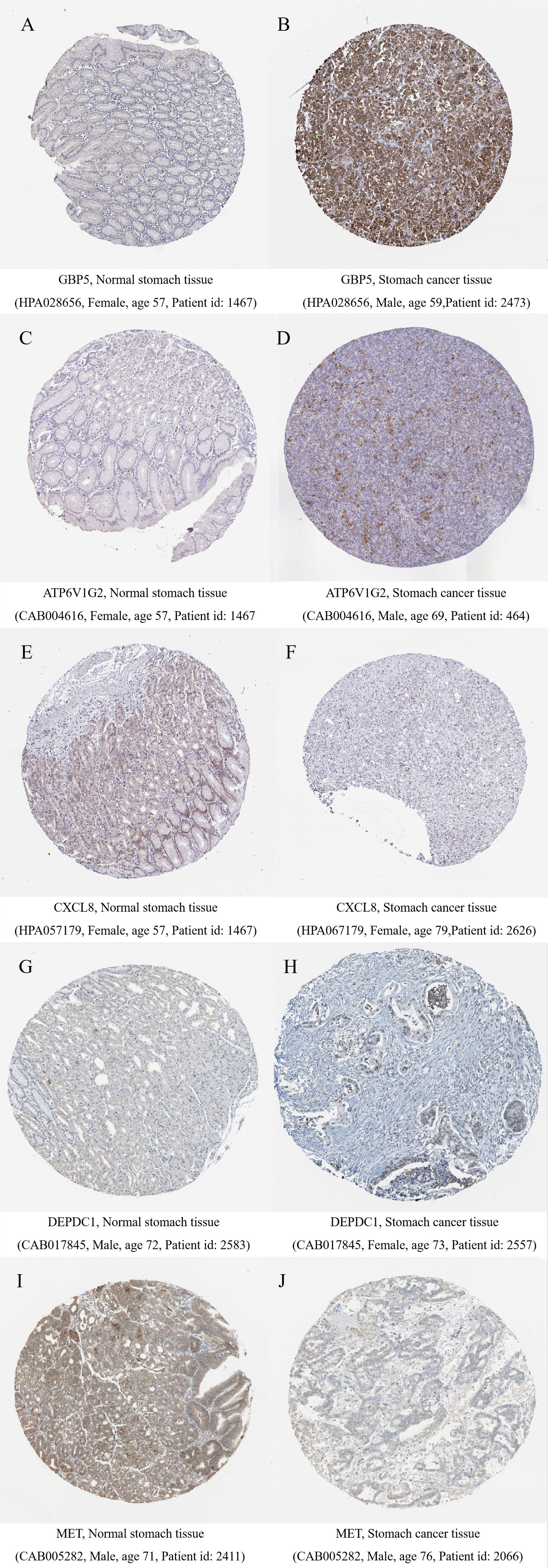

Supplement: Supplementary Figure 2 — Immunohistochemical images from HPA. (A, B) The immunohistochemical images of GBP5 in normal stomach tissues and gastric cancer tissues. (C, D) The immunohistochemical images of ATP6V1G2 in normal stomach tissues and gastric cancer tissues. (E, F) The immunohistochemical images of CXCL8 in normal stomach tissues and gastric cancer tissues. (G, H) The immunohistochemical images of DEPDC1 in normal stomach tissues and gastric cancer tissues. (I, J) The immunohistochemical images of MET in normal stomach tissues and gastric cancer tissues. [file Image_2.jpeg]
